# Comparison between learning curves of robot-assisted and laparoscopic surgery in gynaecology: a systematic review

**DOI:** 10.52054/FVVO.16.4.047

**Published:** 2024-12-27

**Authors:** D Raimondo, A Raffone, D Neola, L de Landsheere, R.A. de Leeuw, L Mereu, T Badotti, E Pazzaglia, R Seracchioli, G Scambia, F Fanfani

**Affiliations:** Division of Gynaecology and Human Reproduction Physiopathology, IRCCS Azienda Ospedaliero-Universitaria di Bologna, 40138 Bologna, Italy; Department of Medical and Surgical Sciences (DIMEC), University of Bologna, 40138 Bologna, Italy; Department of Woman, Child, and General and Specialized Surgery, University of Campania “Luigi Vanvitelli”, 80138 Naples, Italy; Department of Neuroscience, Reproductive Sciences and Dentistry, School of Medicine, University of Naples Federico II, 80131 Naples, Italy; Department of Obstetrics and Gynaecology, CHR de La Citadelle, University of Liège, 4000 Liège, Belgium; Amsterdam University Medical Center, location Vrije Universiteit Amsterdam, Department of Obstetrics & Gynaecology, De Boelelaan 1117, 1081 Amsterdam, the Netherlands; Amsterdam Reproduction and Development, 1081 Amsterdam, the Netherlands; CHIRMED Department of University of Catania, 95123 Catania, Italy; Department of Obstetrics and Gynaecology Policlinico G. Rodolico di Catania, 95123 Catania, Italy; Hospital e maternidade municipal de Sao José dos Pinhais, 83005-040 Sao José dos Pinhais, Brazil; Department of Woman, Children and Public Health Sciences, Gynaecologic Oncology Unit, Fondazione Policlinico Universitario A. Gemelli IRCCS, 00168 Rome, Italy; Institute of Obstetrics and Gynaecology, Catholic University of the Sacred Heart, 00168 Rome, Italy; European Society for Gynaecological Endoscopy (ESGE) Special Interest Group (SIG) on Robotics

**Keywords:** Robotic laparoscopic surgery, gynaecologic surgery, proficiency, learning curve

## Abstract

**Background:**

The advantages and disadvantages of Robotic Laparoscopic Surgery (RLS) compared to other minimally invasive surgical approaches are debated in the literature.

**Objective:**

To evaluate the learning curves (LC) and their assessment methods for Robotic Laparoscopic Surgery (RLS) and Laparoscopic Surgery (LPS) in gynaecologic procedures.

**Materials and Methods:**

A systematic review of the literature was performed including the English language observational or interventional studies reporting the absolute number of procedures needed to achieve competency in RLS and LPS gynaecologic procedures, along with an objective and reproducible LC assessment method.

**Main outcome measures:**

Number of procedures needed to achieve competency in RLS and LPS and LC assessment methods were extracted from included studies.

**Results:**

Six studies with a total of 545 women were included. Several surgical procedures and methods for LC assessment were assessed in the included studies. For radical hysterectomy, bilateral salpingo-oophorectomy and lymph node dissection, the minimum number of procedures required to reach the LC was smaller in RLS than LPS in two studies out of four. For sacrocolpopexy, the number of procedures required to reach the LC was lower in RLS and LPS in one study out of two.

**Conclusion:**

RLS learning curve was reported to be quicker than that of LPS for radical hysterectomy, bilateral salpingo-oophorectomy and lymph node dissection. However, a standardised and widely accepted method for LC assessment in endoscopic surgery is needed, as well as further randomised clinical trials, especially involving inexperienced surgeons.

**What is new?:**

This study may be the first systematic review to evaluate the LCs and their assessment methods for RLS and LPS in gynaecologic procedures

## Introduction

Robotic Laparoscopic Surgery (RLS) has spread more and more worldwide in recent years, spanning 69 countries and involving 6,730 certified surgeons worldwide (Bottura et al., 2022). Over time, RLS has gained increasing attention and has been compared in multiple studies to the conventional minimally invasive surgical approach of laparoscopy (LPS). The integration of RLS in the field of gynaecology represents a significant milestone and allows the development of new surgical techniques. This approach has several advantages, including ergonomic benefits for surgeons, augmented visual acuity, nullified hand tremors, and easier manipulation of instruments (Liu et al., 2022).

Nowadays, RLS can be used to treat both benign and malignant diseases in gynaecology, and the advantages and disadvantages compared to other minimally invasive surgical approaches are a topic of discussion in scientific literature (Aarts et al., 2015; Truong et al., 2016; Gitas et al., 2022; Lee et al., 2022; Liu et al., 2022; Nobbenhuis et al., 2023).

Thus far, gynaecologic RLS data have demonstrated feasibility, safety, and equivalent clinical outcomes compared with LPS and with better clinical outcomes than laparotomy (Aarts et al., 2015), at the cost of longer operative times.

Other advantages of RLS compared to laparotomy include smaller incisions, reduced morbidity, diminished postoperative pain, and shorter hospital stays, as well as LPS.

In surgery, one pivotal element for validating the reproducibility and efficacy of a procedural method is the Learning Curve (LC): this concept delineates how mastery of knowledge or skill is attained through repetition (Schermerhorn et al., 2021). When learning a new procedure, performance improves with experience, and graphically plotting performance against experience produces an LC (Hopper et al., 2007). Therefore, LC can be described as the duration, amount of feedback and repetition needed to achieve a certain predefined level of expertise. In contemporary medical education, the quest for the shorter LC is important, primarily for optimising efficiency and diminishing morbidity. The outcome of a learning curve assessment can be a number, while assessment itself is usually defined as the way the proficiency is examined.

However, the assessment of the LC in surgery is still debatable in literature and, despite some standardised and reproducible methods that have been described, a widely accepted method to define the exact number of procedures a surgeon needs to perform to become competent/proficient is missed.

In this context, this study aims to systematically review the different methods of assessment of LC in gynaecologic surgery and to evaluate the differences in the LC between laparoscopic and robotic approaches.

## Materials and methods

### Study protocol

Each step of this systematic review was defined a priori and described in a protocol registered in PROSPERO international database of prospectively registered systematic reviews (ID: CRD42023391388). All review stages, including search strategy, study selection, risk of bias assessment, data extraction, and data analysis, were independently performed by two authors (D.N and E.P). In case of disagreement, consensus was achieved by discussion among all authors.

The Preferred Reporting Item for Systematic Reviews and Meta-analyses (PRISMA) statement and checklist were adopted for reporting the whole study (Moher et al., 2016).

### Search strategy

The search strategy consisted of searching 5 electronic databases: MEDLINE, Web of Sciences, Scopus, Google Scholar and ClinicalTrial.gov, from their inception to September 2023. We searched the following terms: ((LEARNING CURVE) OR (LEARN*) OR (TRAIN*) OR (IMPROV*) OR (COMPETENCY) OR (SKILL) OR (YOUNG)) AND (((ROBOTIC) OR (ROBOT-ASSISTED) OR (ENDOSCOP*) OR (LAPAROSCOP*) OR (MINI-INVASIVE)) AND ((GYNECOL*) OR (GYNECOLOGICAL SURGERY) OR (HYSTERECTOMY) OR (LYMPHADENECTOMY) OR (ENDOMETRIOSIS))).

References list from each eligible study were also screened for missed studies.

### Study selection

All peer-reviewed studies that compared the LC of gynaecologic procedures in RLS or LPS were included. In particular, we included the English language observational or interventional studies or randomised-controlled clinical trials that reported the minimum number of procedures necessary to achieve competency in both RLS and LPS gynaecologic procedures, which also reported an objective and reproducible method for assessing LC.

Literature reviews, case series, case reports, video articles and studies in languages other than English were a priori considered as exclusion criteria.

### Data extraction

Data extraction was performed without modification of the original data. For each included study, we collected the following data:

- type of study (observational or interventional design, retrospective or prospective data, randomised or non-randomised allocation of patients);
- gynaecologic disease (benign or malignant);
- number of operators;
- competence and experience of the operator/s;
- type of procedure analysed in the study;
- statistical method used to assess the LC
- outcomes considered in LC assessment (i.e. total operative time, estimated blood loss, days of hospitalisation, number of lymph nodes collected);
- absolute minimum number of procedures necessary to reach the LC, (i.e. the minimum number of procedures necessary to achieve competency in that procedure);
- number of patients who have undergone surgery;
- patients age;
- patients Body Mass Index (BMI);
- total surgery time;
- Estimated Blood Loss (EBL);
- postoperative complications;
- length of hospital stay.

### Risk of bias within studies assessment

The Methodological Index for Non-Randomised Studies (MINORS) (Slim et al., 2003) was used to assess the risk of bias within studies. We assessed each included study for 7 applicable domains related to risk of bias: 1) Aim (i.e. if the study had a clearly stated aim); 2) Patient selection (i.e. if patients were randomly or consecutively selected for inclusion in the study); 3) Prospective data collection (i.e. if data collection followed an a priori defined study protocol with an appropriate interval of time between procedures); 4) Appropriate endpoints (i.e. if the assessment of LC was present); 5) Unbiased assessment of the study endpoint (i.e. if the method of assessment of LC was clearly described, objective and reproducible); 6) Appropriate follow-up period (i.e. if the follow-up was at least one month); 7) Loss to follow-up (i.e. if patients lost to follow-up were less than 5% of total study population).

Authors judged each study at “low risk” if data about the domain were “reported and adequate”, “unclear risk” if data about the domain were “not reported”, or “high risk” of bias if data about the domain were “reported but inadequate”.

## Results

### Study selection

After the database searches, 20,853 studies were identified. Duplicate removal and title screening processes led to 2,217 and 128 studies, respectively. Abstract screening led to 23 studies which were evaluated for eligibility. Of them, 17 studies were excluded:

- one study because of data overlapping with another included study (Lim et al., 2010)
- two studies because they are reviews of the literature (Kho, 2011; Vetter et al., 2015)
- 12 studies because they did not assess LC for robotic procedures (Fanning et al., 2008; Feuer et al., 2009; Schreuder et al., 2010; Kilic et al., 2012; Madhuri et al., 2012; Chong et al., 2013; Eddib et al., 2013; Catchpole et al., 2016; Luciano et al., 2016; Sinha et al., 2019; Eoh et al., 2020; Bottura et al., 2022)
- one study because it did not report an absolute number of procedures to reach the LC (Lopez et al., 2016)
- one study because it did not report data about gynaecologic procedures (Schermerhorn et al., 2021)

**Figure 1:**
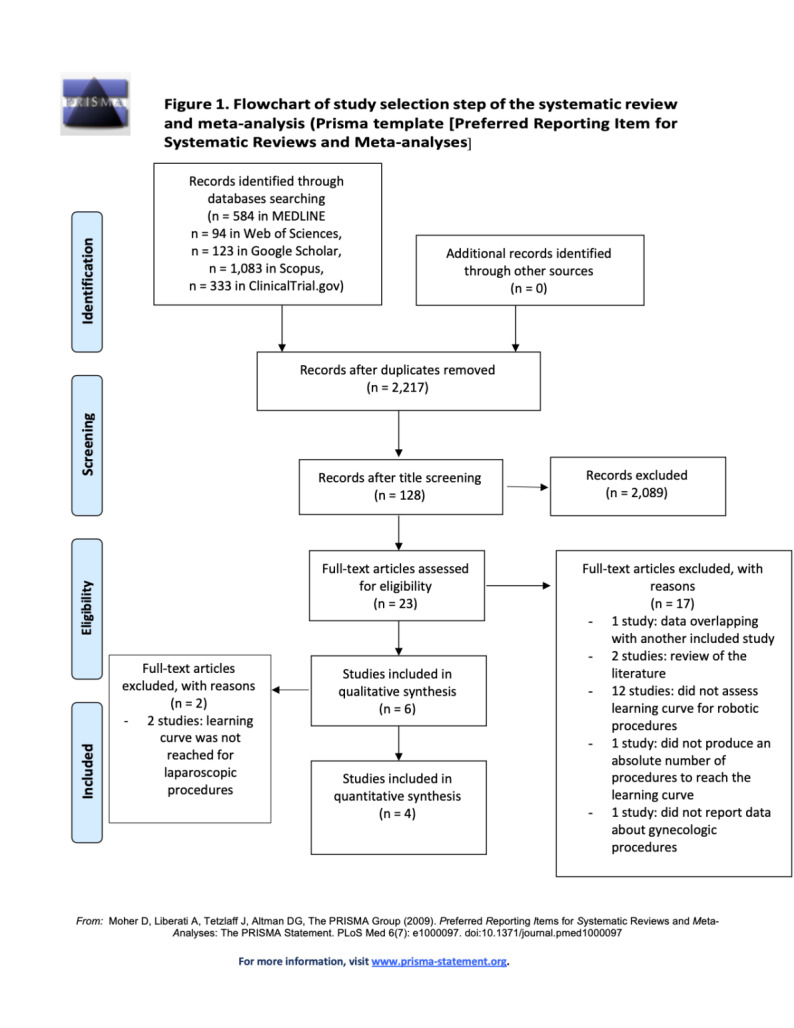
Flowchart of study selection step of the systematic review and meta-analysis according to PRISMA guidelines.

Finally, six studies were included in the qualitative synthesis (Lim et al., 2011; Pulliam et al., 2012; Li et al., 2016; Pilka et al., 2017; Torng et al., 2017; Heo et al., 2018) (Figure 1).

### Studies and patients’ characteristics

A total of 545 women were included in our systematic review: 254 had undergone RLS and 291 had undergone LPS. All included studies were observational, retrospective, cohort studies (Table I).

**Table I:** Characteristics of the included studies.

Study	Type of study	Number of patients, n (%)	Condition (benign or malignant)	Number of operators	Characteristics of operators	Procedure
Lim 2011	Retrospective cohort	244 (44.78)	Malignant (endometrial carcinoma)	1	Surgeon with minimal laparoscopic training	Hysterectomy with LND
Pulliam 2012	Retrospective cohort	91 (16.7)	Benign	3	Fellowship-trained urogynecologists with previous laparoscopic experience	Sacrocolpopexy, sacral hysteropexy, and sacrocervicopexy
Li 2016	Retrospective cohort	61 (11.2)	Malignant (cervical carcinoma)	1	Experienced laparoscopist	Radical hysterectomy and PLND
Pilka 2017	Retrospective cohort	64 (11.7)	Benign	nr	nr	Sacrocolpopexy
Torng 2017	Retrospective cohort	44 (8.1)	Malignant (endometrial carcinoma)	1	Experienced laparoscopist	Hysterectomy with LND
Heo 2018	Retrospective cohort	41 (7.5)	Malignant (cervical carcinoma)	1	nr	Radical hysterectomy with PLND

LND: Lymph node dissection, PLND: Pelvic lymph node dissection, nr: not reported.

Four studies reported data about the comparison of RLS vs LPS for treating malignant conditions (Lim et al., 2011; Li et al., 2016; Torng et al., 2017; Heo et al., 2018). In particular, two studies included women with endometrial carcinoma (Lim et al., 2011; Torng et al., 2017) and two studies included women with uterine cervix carcinoma (Li et al., 2016; Heo et al., 2018). On the other hand, two studies reported data about women with benign conditions (Pulliam et al., 2012; Pilka et al., 2017).

Regarding the surgical procedures, RLS and LPS LC were compared for radical hysterectomy, bilateral salpingo-oophorectomy and lymph node dissection (pelvic or para-aortic) in four studies (Lim et al., 2011; Li et al., 2016; Torng et al., 2017; Heo et al., 2018), and for sacrocolpopexy in two studies (Pulliam et al., 2012; Pilka et al., 2017).

Surgery was performed by experienced laparoscopic surgeons in three studies (Li et al., 2016; Pilka et al., 2017; Torng et al., 2017), by three fellowship-trained urogynaecologists with previous laparoscopic experience in one study (Pulliam et al., 2012), and by a surgeon with minimal laparoscopic training in one study (Lim et al., 2011).

For the assessment of the LC, one study used the cumulative summation technique (CUSUM) (Heo et al., 2018); two studies analysed the presence of a statistical difference between cases before and after an absolute number of procedures (Lim et al., 2011; Li, Du and Jiang, 2016) which was calculated with a “two stages regression line method” (Lim et al., 2011) and with the visualisation of a “turning point” in the LC (Li et al., 2016), respectively; one study used a mathematical method called “moving average technique” (Pulliam et al., 2012); one study used a “generalised additive model” based on Pearson’s correlation test (Torng et al., 2017); one study used the visualisation of plateau of LC (Pilka et al., 2017) (Table II).

**Table II:** Characteristics of the included studies and learning curve assessment.

Study	Characteristics of surgeons	Surgical procedure	Method to assess the LC	Outcomes considered in LC assessment	Number of procedures to reach the LC	
					RLS	LPS
Lim 2011	Surgeon with minimal laparoscopic training	Hysterectomy with LND	Two stages regression line + statistical difference between case series	Operative time	24	49
Pulliam 2012	Fellowship-trained urogynecologists with previous laparoscopic experience	Sacrocolpopexy, sacral hysteropexy, sacrocervicopexy	Moving average technique (moving block technique)	Operative time, Setup time	10	nr
Li 2016	Experienced laparoscopist	Radical hysterectomy and PLND	Statistical difference between case series	Operative time, EBL, peritoneal drainage of first 24 hours	13	10
Pilka 2017	nr	Sacrocolpopexy	Gross visualization of plateau of LC	Operative time	10	10
Torng 2017	Experienced laparoscopist	Hysterectomy with LND	Generalized additive model based on pearson correlation test	Operative time	6	nr
Heo 2018	nr	Radical hysterectomy with PLND	CUSUM	Operative time	13	12

RLS: Robotic laparoscopic surgery, LPS: Laparoscopic surgery, LC: Learning curve, NR: Not reached, LND: Lymph node dissection, PLND: Pelvic lymph node dissection, nr: not reported, CUSUM: cumulative sum control chart.

For radical hysterectomy, bilateral salpingo- oophorectomy and lymph node dissection, the minimum numbers of procedures required to reach the LC were 24 (Lim et al., 2011), 13 (Li et al., 2016), 6 (Torng et al., 2017) and 13 (Heo et al., 2018) in RLS and 49 (Lim et al., 2011), 10 (Li et al., 2016), and 12 (Heo et al., 2018) in LPS. For sacrocolpopexy, the number of procedures required to reach the LC was 10 (Pulliam et al., 2012) and 10 (Pilka et al., 2017) in RLS, while it was 10 in LPS (Pilka et al., 2017). In Torng et al. (2017) and in Pulliam et al. (2012), the LC in LPS was not reached, therefore it was not reported an absolute number of procedures to reach the LC.

Other characteristics of the included studies are shown in Table III.

**Table III:** Characteristics of patients and surgical data.

Study	Patients, n (%)		Age, mean + SD		BMI, mean ± SD		Surgical time in minutes, mean ± SD		EBL, mean ± SD		Postoperative Complications, n (%)		Lenght of stay, mean ±	
	RLS	LPS	RLS	LPS	RLS	LPS	RLS	LPS	RLS	LPS	RLS	LPS	RLS	LPS
Lim 2011	122 (50)	122 (50)	62.1 ± 8.4	61.6 ± 11.8	31 ± 8.8	29.9 ± 7.0	147.2 ± 48.2	147.2 ± 48.2	81.1 ± 45.9	207.4 ± 109.4	1 pulmonary embolism, 2 pelvic abscess, 1 perforated gastric ulcer, 1 perforated bowel, 6 urinary tract infections, 1 superficial vein thrombosis	3 transfusion, 3 obturator nerve palsy, 2 cystotomy repair, 1 enterotomy, 1 venotomy, 1 deep vein thrombosis, 1 vescicovaginal fistula, 1 bacteremia, 1 Richter’s hernia, 1 urinary tract infection, 1 cellulitis	1.5 ± 0.9	3.2 ± 2.3
Pulliam 2012	43 (47.3)	48 (52.7)	59 ± 11.54	59 ± 11.54	26 ± 4	28 ± 5	242 ± 54	242 ± 54	83 ± 78	91 ± 27	1 hemorrage, 1 bladder suture	2 bladder sutures, 1 ureteral kinking	1.75 ± 0.88	2 ± 1.2
Li 2016	37 (60.6)	24 (39.4)	N/A	N/A	N/A	N/A	215.84 ± 56.28	215.84 ± 56.28	309.73 ± 227.80	325.00 ± 220.67	2 hemorragies, 1 ureteral injury	No complications	10 ± 3.19	10.29 ± 3.3
Pilka 2017	13 (20.3)	51 (79.7)	59 ± 11.54	25.5 ± 3.49	25.8 ± 2.75	186.5 ± 19	236.5 ± 76.8	78.5 ± 54.87	119 ± 98.2	N/A	N/A	N/A	N/A	N/A
Torng 2017	20 (45.5)	24 (54.5)	56.4 ± 7.7	57.0 ± 5.5	26.1 ± 7.3	24.6 ± 5.1	286.9 ± 76.7	286.9 ± 76.7	233.5 ± 430.2	122.5 ± 110.2	1 blood transfusion, 1 bladder perforation, 1 vaginal cuff dehiscence	1 obturator nerve injury, 1 conversion to laparotomy	3.9 ± 1.6	3.8 ± 1.3
Heo 2018	19 (46.3)	22 (53.7)	47.4 ± 10.3	54.5 ± 13.3	23.8 ± 4.4	24.0 ± 2.9	316.1 ± 76.9	316.1 ± 76.9	468.4 ± 312.8	436.4 ± 201.3	2 post-op bleeding, 1 peritonitis, 1 dehiscence of trocar site	6 void difficulty, 1 postoperative bleeding, 1 right basal ganglia infarction, 1 fever	12.1 ± 5.1	19 ± 17.5
Total	254 (46.6)	291 (54.4)									24 (9.4)	29 (10)		

BMI: Body Mass Index, EBL: Estimated blood loss, nr: not reported

## Discussion

### Main findings and interpretation

This study shows that the LC of gynaecologic minimally invasive procedures is assessed through different methods in the literature and was reported to be quicker in RLS than that in LPS for radical hysterectomy, bilateral salpingo-oophorectomy and lymph node dissection in two out of four included studies, and for sacrocolpopexy in one included study out of two.

Although the LC in RLS has been reported to be quicker when compared to traditional LPS for most of the common minimally invasive procedures, these findings lack consistent validation on a larger scale due to variations in the definitions of LC and diverse clinical outcome measures employed across the studies assessing gynaecologic minimally invasive procedures.

When discussing medical training, the words “competency”, “proficiency” and efficiency have specific meanings. “Competency” describes the ability to perform a task required for a work situation (e.g. lower operative time in hysterectomy), “proficiency” describes the ability to perform a task with skill, impacting clinical outcomes (e.g. lower recurrence rate of cancer) and “efficiency” describes the maximum ability to perform a task with skill, with no further improvement observed over time (steady state). In detail, some studies define the LC as the number of cases required to stabilise operative time to perform the various procedures. In contrast, other authors considered how the surgeons’ skills affected the clinical outcomes of the patient (Baeten et al., 2021). Yet, other researchers took a more comprehensive approach that correlated some outcomes such as perioperative complications, operative time, and length of hospital stay, with surgical diagnosis, procedure performed and surgeon experience (Bottura et al., 2022). Liu et al. (2022) focused on evaluating the plateauing of the surgical procedure time and time for robot docking and port placement. Lee et al. (2022) adopted a similar approach and applied the cumulative summation technique (CUSUM), which is a sequential analysis developed by E. S. Page of the University of Cambridge to monitor the success and failure of a technical skill and trends.

With particular regard to gynaecologic RLS, the choice of a methodology for evaluating the LC also varies among the studies, reflecting the complexity of comparing skill acquisition timing. In particular, each study included in this systematic review used a different method of assessment of LC.

This heterogeneity in LC assessment highlights the need to establish a consistent, standardised, and reproducible method to assess the LC of gynaecologic minimally invasive surgical procedures. Such a standardised LC assessment might positively affect education, training, competency and proficiency of new endoscopic surgeons. Despite the methodological heterogeneity prevalent in the field and the consequent difficult comparison of the studies, the existing literature suggests a faster LC in RLS than LPS. Our analysis seems to confirm these findings. The study by Li et al. (2016) reported a quicker learning curve for LPS than that for RLS. This finding may be explained by using 3D visualisation in LPS, which provides better perception of depth and spatial resolution than 2D LPS, comparable to the visualisation system of RLS. Indeed, it was demonstrated that 3D LPS laparoscopy appears to improve speed and reduce the number of performance errors when compared to 2D LPS (Sørensen et al., 2016), and allows trainees to reach proficiency sooner in simulators (Ashraf et al., 2015). Further studies appear needed to assess differences between LCs in 3D LPS and RLS exclusively.

The difference between LCs in RLS and LPS might be even more marked since the competency in the LC was not reached in LPS, while a plateau in LC was reached in RLS (Pulliam et al., 2012; Torng et al., 2017). Yet, LC of RLS might be further improved by the future development of surgical instruments and training programs and mentorship.

However, LPS and open surgery should not be neglected as evidence from other surgeries suggests that prior experience in LPS seems to improve perioperative outcomes for surgeons when starting with RLS (Harke et al., 2022). Indeed, studies assessing LCs on simulators showed that having a previous laparoscopic training may improve performance in RLS simulator tasks, suggesting a transference of skills from laparoscopic to robotic surgery (Davila et al., 2018; Kanitra et al., 2021). Furthermore, complications during RLS could require conversion to LPS or open surgery. Thus, adequate LPS (and open surgery) training might be recommended before approaching RLS to guarantee a safe surgery for patients.

### Strengths and limitations

To our knowledge, this may be the first systematic review to evaluate the different LC assessment methods in gynaecologic minimally invasive surgery and to compare the LC in laparoscopic and robotic gynaecologic surgery. Moreover, our findings might be supported by a good overall quality of the included studies as shown by the risk of bias within studies assessment.

However, some limitations may affect our study, such as the retrospective design of the included studies, the low number of patients included, the non-homogeneity of the LC evaluation methods and the adoption of operators with experience in LPS in five out of six included studies. In particular, LCs in RLS of surgeons who already have experience in LPS may appear quicker, since such surgeons are already familiar with endoscopic vision of the pelvis, instrumentation and surgical procedure steps. Nonetheless, the included studies reported a descriptive and subjective characterisation of operators’ surgical experience (i.e., minimal, fellowship-trained, experienced), rather than the exact number of procedures performed before the LC assessment. Anyway, the adoption of unskilled laparoscopic surgeons might also increase the difference in LC in favour of RLS.

Moreover, our findings may be affected by heterogeneity in the complexity of the procedure, the intensity of the period of learning, and the outcome chosen to evaluate competency or proficiency. However, in each included study comparison between RLS and LPS was homogeneous in terms of surgical procedure, surgeon’s experience, intensity of period of learning and outcomes chosen to assess LCs.

## Conclusion

The LC of gynaecologic minimally invasive procedures is assessed through different methods in the literature, highlighting the need for the development of a standardised method. However, despite the inhomogeneity of LC assessment in the included studies, RLS LC was reported to be quicker than that of LPS for radical hysterectomy, bilateral salpingo-oophorectomy and lymph node dissection and for sacrocolpopexy in three out of six studies, and equal in one study. Additional studies with unskilled surgeons are needed to further explore this field.
